# Right intra lobar pulmonary sequestration with feeding artery arising from abdominal aorta: a case report

**DOI:** 10.1186/s13019-015-0290-1

**Published:** 2015-06-25

**Authors:** Satoshi Fumimoto, Kaoru Ochi, Yoshio Ichihashi, Kiyoshi Sato, Takuya Morita, Nobuharu Hanaoka, Takahiro Katsumata

**Affiliations:** Department of Thoracic and Cardiovascular Surgery, Osaka Medical College, Osaka, 2-7 Daigakumachi, Takatsuki, Osaka 569-8686 Japan

**Keywords:** Intra lobar sequestration, Video-assisted thoracic surgery, Aberrant artery

## Abstract

Pulmonary sequestration (PS) is a rare congenital malformation. Right intra lobar PS with a feeding artery arising from the abdominal aorta is extremely rare. This case report describes a 30-year-old man with a history of mental deficiency and repeated pneumonia who was referred to our hospital for further work-up of PS. Three-dimensional enhanced computed tomography of the chest and aorta revealed right intra lobar PS with an aberrant systemic artery from the abdominal aorta. We resected the PS using lower lobectomy by video-assisted thoracic surgery (VATS). The patient was discharged 10 days later without complications.

## Background

Pulmonary sequestration (PS) is an uncommon congenital malformation of the foregut, usually characterized by nonfunctional lung tissue separated from the normal tracheobronchial tree and fed by an aberrant systemic artery. PS accounts for 0.15 % to 6.45 % of all pulmonary malformations [1]. Despite being a benign condition, the potential complications of PS are serious and may include recurrent pulmonary infection, hemoptysis, congestive heart failure, and tumorigenesis. For this reason, the main form of treatment has always been surgical excision even for asymptomatic patients with PS.

## Case presentation

**Fig. 1:**
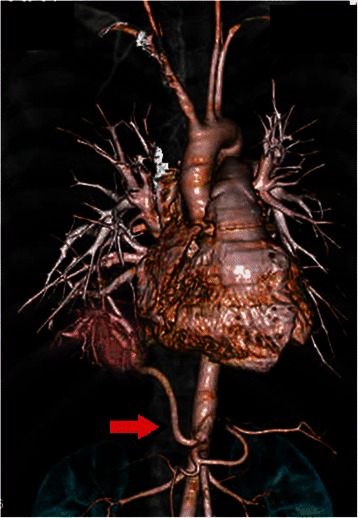
Computed tomography of the chest and aorta reveals right PS with aberrant systemic artery arising from the abdominal aorta

The patient, a 30-year-old man with a past history of mental deficiency and repeated pneumonia, was referred to our hospital for further evaluation of PS which was identified when he received a health diagnosis. He had no history of smoking. A physical examination and laboratory investigation showed no specific findings. Three-dimensional enhanced computed tomography of the chest and aorta revealed intra lobar PS with an aberrant systemic artery originating from the abdominal aorta, and flowing into a consolidation lying in the posterior basal segment of the right lower lobe (Fig. 1). The bronchus, pulmonary artery, and pulmonary vein of the right lower lobe appeared normal on the computed tomographic scan. On the basis of these findings, the patient was diagnosed with intra lobar PS, which was of type 1 according to Pryce’s classification, and treatment using video-assisted thoracic surgery (VATS) was planned.

**Fig. 2:**
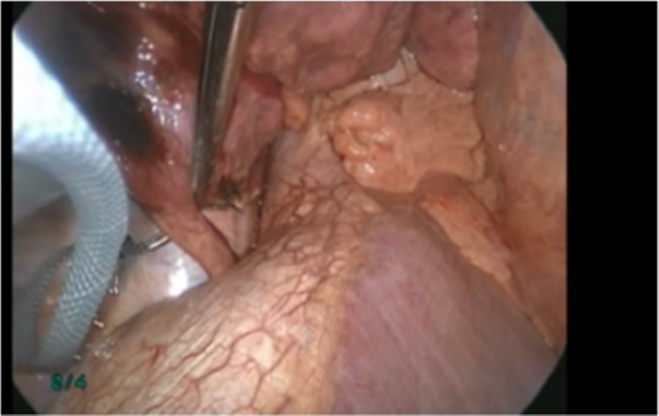
An operative photo shows the aberrant artery (7 mm in diameter) running parallel to the inferior vena cava flowing into the lower lobe

**Fig. 3:**
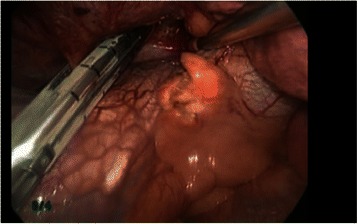
The aberrant artery is transected using a single stapling device at a distance 2 cm from the diaphragm toward the head

The patient was administered general anesthesia using one-lung ventilation and was placed in a full left lateral decubitus position. A 10-mm 30-degree thoracoscope was inserted into the right pleural cavity through a 3-cm incision in the 7th intercostal space along the midaxillary line, at which time several capillaries meandering on the visceral pleura of the lower lobe were found. Running parallel to the inferior vena cava, the aberrant artery (7 mm in diameter) flowing into the lower lobe was identified (Fig. 2). A 4-cm incision in the 5th intercostal space along the anterior axillary line and a 3-cm incision in the 7th intercostal space along the posterior axillary line were made. The aberrant artery was transected using a 45-mm stapling device at a distance of 2 cm from the diaphragm toward the head (Fig. 3) and a standard VATS lobectomy was performed The patient had no postoperative complications and was discharged on postoperative day 10.

## Discussion

As a rare congenital malformation of the lower respiratory tract, PS lacks communication with the tracheobronchial tree and receives an aberrant arterial blood supply from the systemic circulation. The term ‘sequestration’ was first used to describe PS by Pryce in 1946 [2]. This condition is divided into two types: intra lobar PS and extra lobar PS. Pryce further subdivided intra lobar PS into three types. In type 1, there is no abnormal lung tissue, and the artery of the systemic circulation enters the lung with the normal bronchus. In types 2 and 3, there is abnormal lung tissue, and blood supply from the anomalous artery either reaches the normal lung or is confined to the sequestered lung. In our case, the image findings led us to the diagnosis of type 1 intra lobar PS, with an aberrant artery arising from the abdominal aorta. With respect to the aberrant source of pulmonary sequestration, Sun and colleagues reported 62 (86.1 %) aberrant arteries originating from the thoracic aorta, 5 (6.9 %) from the abdominal aorta, 4 (5.6 %) from the phrenic artery, and 1 (1.4 %) the intercostal artery among the 72 patients in their study [3]. A similar trend has been described in other reports, in which branching from the abdominal aorta was rare compared with that from the thoracic aorta. When lesions are further separated by left and right side, the number of cases with lesions on the right side is lower. To the best of our knowledge, PS resection with VATS for right intra lobar PS with feeding artery arising from the abdominal aorta has not been reported. Compared with the conventional posterolateral thoracotomy approach, the most important step during resection of PS via VATS is the identification of the aberrant artery [4]. The aberrant artery may be thickened or fragile because of the recurrent infections. If unanticipated injury to the aberrant artery occurs, bleeding cannot be effectively managed because of high blood pressure.

Preoperative imaging helped us anticipate the location of the target vessel. Aortography was once considered to be the gold standard for the diagnosis of PS, as it explicitly reveals the aberrant arterial supply. However, with the advent of noninvasive techniques, the aberrant artery in PS can now be clearly identified on coronal and three-dimensional reconstructed images obtained using computed tomography [5]. Another point to be emphasized regarding the VATS is that the proximal end should be long enough for the introduced stapling device to cut the aberrant artery. If transection of the aberrant artery is performed as close to the lung tissue as possible at the beginning to preserve a relatively long proximal end, we can easily manage the excessive bleeding if vascular injury occurs. Finally, for transection of the aberrant artery, we emphasize the way of using the stapling device. Despite using VATS, some surgeons still choose not to staple the aberrant artery using a single stapling device [6]. Kestenholz and colleagues described occluding the artery centrally with a stapling device after removal of the endoscopic scalpel. They then cut the artery peripherally with a second stapling device. With experience, however, they recognized that this method was unnecessary and began using a single stapling device even with very large vessels. Seok also reported the experience transecting a large feeding artery arising from the descending thoracic aorta using only one stapling device safely [7].

Minimal access trauma by VATS involves less postoperative pain, in addition to evidence suggesting better preservation of postoperative lung function, compared with posterolateral thoracotomy [8]. In view of these points, we propose that the VATS approach for surgically treating PS is safe and of great benefit to patients.

## Conclusions

Unlike conventional lobectomy, PS resection requires the identification and dissection of the aberrant artery. However, if performed as described here, VATS is possible and useful for surgically treating PS.

## Consent

Written informed consent was obtained from the patient for publication of this case report and all accompanying images.

## Abbreviations

PS: Pulmonary sequestration
VATS: Video-assisted thoracic surgery
